# Gender differences in health-related quality of life of Australian chronically-ill adults: patient and physician characteristics do matter

**DOI:** 10.1186/1477-7525-11-102

**Published:** 2013-06-21

**Authors:** Upali W Jayasinghe, Mark F Harris, Jane Taggart, Bettina Christl, Deborah A Black

**Affiliations:** 1 Centre for Primary Health Care and Equity, University of New South Wales, Sydney, New South Wales, Australia; 2 Westmead Breast Cancer Institute, Westmead, New South Wales, Australia; 3 Faculty of Health Sciences, University of Sydney, Lidcombe, New South Wales, Australia

**Keywords:** Quality of life, Patient and physician characteristics, SF-12 version 2, Physical component score, Mental component score, Multilevel regression analysis

## Abstract

**Background:**

The aims of this study were to explore the health-related quality of life (HRQoL) in a large sample of Australian chronically-ill patients (type 2 diabetes and/or hypertension/ischaemic heart disease), to investigate the impact of characteristics of patients and their general practitioners on their HRQoL and to examine clinically significant differences in HRQoL among males and females.

**Methods:**

This was a cross-sectional study with 193 general practitioners and 2181 of their chronically-ill patients aged 18 years or more using the standard Short Form Health Survey (SF-12) version 2. SF-12 physical component score (PCS-12) and mental component score (MCS-12) were derived using the standard US algorithm. Multilevel regression analysis (patients at level 1 and general practitioners at level 2) was applied to relate PCS-12 and MCS-12 to patient and general practitioner (GP) characteristics.

**Results:**

Employment was likely to have a clinically significant larger positive effect on HRQoL of males (regression coefficient (B) (PCS-12) = 7.29, P < 0.001, effect size = 1.23 and B (MCS-12) = 3.40, P < 0.01, effect size = 0.55) than that of females (B(PCS-12) = 4.05, P < 0.001, effect size = 0.78 and B (MCS-12) = 1.16, P > 0.05, effect size = 0.16). There was a clinically significant difference in HRQoL among age groups. Younger men (< 39 years) were likely to have better physical health than older men (> 59 years, B = −5.82, P < 0.05, effect size = 0.66); older women tended to have better mental health (B = 5.62, P < 0.001, effect size = 0.77) than younger women. Chronically-ill women smokers reported clinically significant (B = −3.99, P < 0.001, effect size = 0.66) poorer mental health than women who were non-smokers. Female GPs were more likely to examine female patients than male patients (33% vs. 15%, P < 0.001) and female patients attending female GPs reported better physical health (B = 1.59, P < 0.05, effect size = 0.30).

**Conclusions:**

Some of the associations between patient characteristics and SF-12 physical and/or mental component scores were different for men and women. This finding underlines the importance of considering these factors in the management of chronically-ill patients in general practice. The results suggest that chronically ill women attempting to quit smoking may need more psychological support. More quantitative studies are needed to determine the association between GP gender and patient gender in relation to HRQoL.

## Background

In 2004, 77% of Australians reported having at least one long-term medical condition [1]. The management of chronic illness has become a major focus in general practice in Australia, both because of its prevalence and because of the opportunity for general practice to intervene early to improve quality of life, prevent disability, and reduce hospital use [2].

The SF-36 and SF-12 are widely used measures of health-related quality of life (HRQoL). Investigators from numerous countries representing diverse cultures have determined that both measures are sensitive to differences in a number of socio-demographic and clinical variables, including gender [3,4], age [2,3], income [4-6], employment [2,4,5], education [5,6], marital status [2,7], satisfaction with care [2], smoking [8-10], and number of chronic conditions [6]. Several studies have found women to have lower HRQoL scores than men [11,12] and a few studies have found that smoking had a greater negative effect on HRQoL of women than men [13]. Employment had a stronger association with the quality of life of males than that of females [2]. In addition to patient factors, we also included the socio-demographic characteristics of the general practitioners (GPs). Earlier research has shown that female GPs provided longer consultations and saw patients with different health problems than their male colleagues [14,15]. Gender is one of the many factors that impact upon the doctor–patient interaction [16]. From the above findings, patient and GP characteristics tended to interact with gender of patients in predicting HRQoL, so we decided to analyse data separately for males and females.

In this study, we investigated the relationship between patient or GP characteristics and HRQoL in a large sample of chronically-ill Australian adults from two states and the Australian Capital Territory, using SF-12 version 2. No one we know of has investigated the impact of characteristics of general practitioners on their patients’ HRQoL before. Based on the findings and identified gaps in the literature, the following research questions were posed:

1) What are the differences in patient gender among subgroups of patient age, socio-economic status (home and car ownership, education and employment), marital status, smoking status, number of chronic diseases, satisfaction with care, GP gender, country of graduation, age (years as a GP), years in the current practice, GP’s practice size and location?

2) Are these factors independent predictors of HRQoL for male and female patients?

3) Are there any clinically significant differences in HRQoL among patient and GP subgroups?

## M**ethods**

### Participants

The Teamwork study was a cluster randomised trial involving 60 practices in urban and rural New South Wales (NSW), Victoria and the Australian Capital Territory between July 2006 and June 2008 [17]. This study aimed to evaluate the impact of facilitating an enhanced role for non-GP staff in the management of patients with chronic illness. Chronic illnesses are diseases of long duration and generally slow progression. In this study we included chronic ill patients suffering from three conditions (type 2 diabetes and/or hypertension/ischaemic heart disease) for more than three months duration as diagnosed by the GP. In the Teamwork study, 3349 type 2 diabetes and/or hypertension/ischaemic heart disease patients aged 18 years or more attending 60 practices were invited by their GPs to participate, and of these, 2642 (79%) consented to participate. Patients were eligible to participate in the study if they had attended the practice in the preceding 12 months. For 2181 (1018 males and 1163 females) of the participating patients, their GPs (n=193) had completed a clinical care interview (CCI) which provided information on the GP’s socio-demographic and practice characteristics. In this paper, a cross-sectional analysis of baseline data was conducted. Pearson Chi-Squared tests indicated that the proportions of gender, age, employment, education, home and car ownership, marital status, smoking status, and number of conditions were similar between all patients who consented to participate (n = 2642, 97% of whom provided patient characteristics) and the subgroup of patients whose GPs had completed the CCI (n = 2181).

A priori sample size calculation on the SF-12 mental component score predicted that, after adjustment for clustering, an average sample size of 30 patients from each of the 60 practices would have sufficient power (1-β= 0.8 and α= 0.05) to detect an effect size of 0.13 between smokers and non-smokers, assuming that about 10% of the patients were smokers (previous studies on SF-12 indicated a cluster effect (ICC = intra-cluster correlation) of 0.011 for the MCS-12 score [2]). Similarly 10 female patients attending each of 90 male or female GPs would have sufficient power (1-β= 0.8 and α= 0.05) to detect an effect size of 0.15 for physical component scores between male and female GPs.

### Ethics

Ethics approval for the study was obtained from the University of New South Wales (UNSW) Human Research Ethics Committee, and we obtained full informed written consent from participants.

### Instruments

The standard SF-12 version 2 is a 12-item questionnaire measuring physical and mental health [6,18]. Use of the SF-12 version 2 has been recommended in preference to the original version 1 form for all new studies [19]. It is an abbreviated form of the SF-36 Health Survey, which is one of the most widely used instruments for assessing HRQoL [6]. Both instruments produce eight dimensions of health: physical functioning (PF), role limitations due to physical problems (RF), bodily pain (BP), general health (GH), vitality (VT), social functioning (SF), role limitation due to emotional problems (RE), and mental health (MH) [20,21]. They also produce two summary scores – the Physical Component Summary (PCS) and the Mental Health Component Summary (MCS) – and have been validated for use in the USA, UK and many other European countries for large-scale health measurement and monitoring [6,21]. For ease of interpretation, scores are standardized to population norms, with the mean score set at 50 (SD=10); higher scores indicate better health. The SF-12 has been shown to have good validity and reliability [19]. Previous research supports the use of the standard SF-12 in Australian settings, and it has been validated for Australia using standard US-derived scoring of the SF-12 summary scores [2,22,23].

Patients were invited by their GPs to participate and were mailed the SF12 and the Patient Assessment of Chronic Illness Care (PACIC) questionnaire with some additional demographic questions including smoking status and chronic medical condition/conditions. A reminder survey was mailed if there was no response after 4 weeks. The PACIC is an instrument that measures patients’ assessment of the care they receive. The psychometric properties of the PACIC have been evaluated in Australia [17].

### Data and variables

The dependent variables were PCS-12 and MCS-12 scores. The GP characteristics included were gender, country of graduation, number of years in the current practice, number of years as a GP, and practice size and location (urban/rural). Both number of years in the current practice and number of years as a GP were divided into tertiles (low, middle, high values). The socio-demographic characteristics of patients studied were gender, age, home and car ownership, education, employment, marital status, smoking status, number of chronic diseases, and overall satisfaction with care (PACIC) (Table 1). Patients were asked if they were current smokers (“Do you currently smoke?”). Home and car ownership can be considered markers of economic status [24].

**Table 1:** Distribution of patient gender according to patient and general practitioner characteristics for 2181 chronically-ill adults

**Variable (definition)**	**Gender**				
	**Male (n = 1018)**		**Female (n = 1163)**		**p-value**^**1**^
	**Number**	**%**	**Number**	**%**	
*Characteristics of patients*					
Age, years					
18–39	18	1.8	50	4.3	0.002
40–59	274	27.0	322	27.8	
> 59	722	71.2	788	67.9	
Health status					
Good	603	60.4	631	55.4	0.019
Poor	396	39.6	509	44.6	
Home ownership					
Own home and car	795	79.2	797	69.3	<0.001
Own home only	22	2.2	121	10.5	
Own car only	136	13.5	154	13.4	
Rent and do not own a car	51	5.1	78	6.8	
Education					
Degree/Diploma	370	36.6	356	30.9	0.006
Elementary/High school	642	63.4	795	69.1	
Employment					
Employed	356	35.1	329	28.6	<0.001
Retired	512	50.5	507	44.1	
Unemployed^2^	145	14.3	314	27.3	
Marital status					
Married (married/cohabiting)	766	77.8	722	64.1	<0.001
Unmarried^3^	219	22.2	405	35.9	
Disease					
One condition	571	56.7	768	68.3	<0.001
Two or more conditions	436	43.3	357	31.7	
Smoker					
Yes	88	8.8	123	10.8	0.127
No	909	91.2	1016	89.2	
Overall PACIC score					
High (above median)	457	46.2	557	50.8	0.035
Low (below median)	533	53.8	540	49.2	
*Characteristics of general practitioner (GP)(n = 193)*					
Gender of GP					
Male (n = 121)	863	84.8	785	67.5	<0.001
Female (n = 72)	155	15.2	378	32.5	
Country of graduation					
Australia (n = 143)	834	81.9	912	78.6	0.686
Overseas (n = 49)	184	18.1	248	21.4	
Years in the current practice					
<6 (n = 85)	337	33.3	462	40.1	0.001
6–15 (n = 56)	266	26.3	307	26.7	
> 15 (n = 47)	408	40.4	382	33.2	
Years as a GP					
< 19 (n = 77)	288	28.5	427	37.3	<0.001
19–24 (n = 48)	319	31.6	366	32.0	
> 24 (n = 54)	403	39.9	352	30.7	
Works in a small practice (1–3 GPs) (n=39)	435	42.7	487	41.9	0.686
Works in a large practice (>3 GPs) (n=154)	583	57.3	676	58.1	
Location of work					
Urban (n = 101)	503	49.4	524	45.1	0.042
Rural (n = 92)	515	50.6	639	54.9	

Notes: ^1^p-values are for comparison of categories of each variable by gender using Pearson’s chi-square test.

^2^Includes patients looking for work, studying full-time, looking after family and unable to work due to sickness or disability.

^3^Includes single/separated/divorced/widowed.

Unknowns: Age = 7, Home and car ownership = 27, Education = 18, Employment = 18, Marital status = 69, Disease = 50, Smoking = 46, Overall PACIC score = 94, Years as a GP = 26, Years in the practice = 19.

Patient characteristics were collected independently using PACIC for the same respondents.

n = number of GPs.

#### Statistical analyses

Summary physical (PCS-12) and mental (MCS-12) components were constructed using the standard SF-12 version 2 US algorithm which is empirically derived from the data of a US general population survey [19]. The dimensions as documented by Kontodimopoulos et al. [25] and Ware et al. [19] were confirmed and validated for Australia using standard US-derived scoring of the SF-12 summary scores [2].

Univariate analyses were carried out using IBM SPSS version 20 (Tables 1 and 2). First, we examined the association between the independent variables and patient gender using the Pearson chi-squared test. Mean unadjusted scores of PCS-12 and MCS-12 among male and female patients in each category of the independent variables were compared using t-tests.

**Table 2:** Unadjusted mean and standard deviation of PCS-12 and MCS-12 scores by characteristics of practices and patients

	**PCS-12**			**MCS-12**		
**Variable (definition)**	**Male**	**Female**		**Male**	**Female**	
	**Mean (SD)**	**Mean (SD)**	**p-value**^**1**^	**Mean (SD)**	**Mean (SD)**	**p-value**^**1**^
Overall score	43.0(11.7)	42.3(12.4)	0.206	50.0(10.9)	48.3(11.2)	0.002
*Characteristics of patients*						
Age, years						
18–39	49.3(7.7)	46.3(11.3)	0.246	46.7(10.7)	41.9(11.9)	0.154
40–59	46.1(10.7)	45.9(11.9)	0.870	48.2(11.0)	45.3(11.8)	0.005
> 59	41.6(11.8)	40.5(12.4)	0.106	50.8(10.7)	50.0(10.4)	0.214
Health status						
Good	48.1(9.1)	48.3(9.4)	0.822	53.0(8.7)	52.0(9.1)	0.059
Poor	35.2(10.8)	34.5(11.6)	0.397	45.4(12.2)	43.4(11.7)	0.025
Home ownership						
Own home and car	43.8(11.4)	44.1(12.1)	0.605	50.9(10.4)	49.3(10.7)	0.007
Own home only	38.2(11.4)	37.9(12.1)	0.939	47.9(10.8)	47.2(11.4)	0.820
Own car only	42.4(12.1)	39.7(12.3)	0.081	47.1(11.6)	45.7(11.0)	0.323
Rent and do not own a car	34.7(11.5)	34.5(12.5)	0.935	42.3(11.8)	44.5(13.9)	0.423
Education						
Degree/Diploma	45.7(10.3)	44.8(11.9)	0.284	51.7(10.3)	48.0(10.6)	<0.001
Elementary/High school	41.5(12.2)	41.2(12.5)	0.688	49.2(11.1)	48.3(11.4)	0.199
Employment						
Employed	48.0(9.2)	48.2(10.7)	0.785	49.7(9.9)	47.1(10.9)	0.003
Retired	41.5(11.6)	40.5(11.8)	0.224	52.0(10.1)	51.0(9.9)	0.152
Unemployed^2^	35.6(11.9)	38.9(13.2)	0.020	43.7(13.3)	45.3(12.3)	0.248
Marital status						
Married (married/cohabiting)	43.4(11.5)	43.4(12.2)	0.998	50.6(10.5)	49.4(10.8)	0.061
Unmarried^3^	41.4(12.4)	39.9(12.6)	0.163	47.6(12.1)	46.2(11.6)	0.225
Disease						
One condition	45.1(11.6)	43.6(12.4)	0.035	50.3(10.5)	49.0(11.0)	0.052
Two or more conditions	40.3(11.1)	39.5(12.2)	0.378	49.5(11.3)	47.2(11.1)	0.010
Smoker						
Yes	41.3(11.8)	41.0((12.3)	0.850	48.0(11.5)	41.9(12.1)	0.001
No	43.3(11.6)	42.4(12.5)	0.157	50.1(10.8)	49.1(10.8)	0.056
Overall PACIC score						
High (above median)	42.6(11.7)	41.1(12.6)	0.070	50.3(10.7)	48.4(10.6)	0.009
Low (below median)	43.3(11.7)	43.1(12.3)	0.813	49.3(11.1)	47.6(11.6)	0.030
*Characteristics of general practitioner (GP)*						
Gender of GP						
Male	42.8(11.8)	**41.1(12.5)**	0.014	49.9(10.8)	48.5(11.3)	0.017
Female	44.4(10.7)	**44.8(11.9)**	0.716	50.1(11.3)	47.8(11.0)	0.050
Country of graduation						
Australia	42.9(11.8)	42.3(12.5)	0.366	50.4(10.9)	48.4(11.1)	0.001
Overseas	43.8(11.0)	42.4(12.2)	0.292	48.2(10.8)	47.8(11.5)	0.718
Years in the current practice						
<6	42.8(11.1)	43.0(12.2)	0.799	49.3(10.8)	47.3(11.3)	0.028
6–15	42.6(11.8)	41.7(12.9)	0.449	49.4(12.4)	49.6(11.1)	0.853
> 15	43.6(12.1)	42.0(12.1)	0.094	51.0(9.9)	48.5(11.0)	0.003
Years as a GP						
< 19	44.0(11.0)	43.1(12.2)	0.403	49.2(10.7)	48.1(11.3)	0.242
19–24	42.3(12.1)	42.6(12.2)	0.819	50.3(11.0)	48.9(11.0)	0.118
> 24	43.1(11.7)	41.2(12.9)	0.068	50.2(10.8)	47.9(11.6)	0.013
Works in a small practice (1–3 GPs)	42.8(12.4)	41.9(13.0)	0.312	49.0(11.3)	47.5(11.6)	0.082
Works in a large practice (> 3 GPs)	43.2(11.1)	42.6(12.0)	0.437	50.7(10.5)	48.9(10.8)	0.006
Location of work						
Urban	43.7(11.4)	42.2(12.0)	0.096	51.2(9.9)	48.3(11.0)	<0.001
Rural	42.5(11.8)	42.3(12.8)	0.804	49.0(11.5)	48.3(11.3)	0.296

Notes: ^1^p-values are for comparison of difference of each category of patient and practice characteristics using independent t- test.

^2^Includes patients looking for work, studying full-time, looking after family and unable to work due to sickness or disability.

^3^Includes single/separated/divorced/widowed.

Number in each category is as shown in Table 1 subject to small number of missing values in SF12 items.

Unknowns: Age = 5, Home and car ownership = 21, Education = 11, Employment = 10, Marital status = 69, Disease = 50, Overall PACIC score = 52.

Patient characteristics were collected independently using PACIC for the same respondents.

Number of GPs is shown in Table 1.

We computed Cohen’s d effect sizes. Cohen defined an effect size of 0.20 as small, 0.50 as moderate, and 0.80 or greater as large [26]. An effect size of more than 0.5 or half a standard deviation is considered to be clinically significant [2,3].

### Multilevel models

Multilevel regression models were used for male and female patients with two dimensions (physical and mental component scores) as continuous dependent variables and general practice and patient characteristics as independent variables. Multilevel analysis used MLwiN 2.25 [27] adjusted for clustering of patients (level 1) within GPs (level 2) [2,4]. Initially, we fitted a baseline variance component model (no independent variables) for each of the response variables followed by the main model. The main model expands the baseline model by including patient and GP characteristics. Parameter estimates of fixed effects were tested for significance using a *t*-test, determined by dividing the estimated coefficients by their standard errors. The significance of the random variance estimates was assessed using the Wald joint chi-squared test [27].

### Multiple imputations

We used multiple imputations to address the potential bias and loss of precision that could result from complete-case analysis. Multiple datasets were created, as each missing value was replaced with a set of random plausible values conditional on known covariates and known distributional information. REALCOM-IMPUTE software with MLwiN was used for the analysis [28]. A total run length of 6500 iterations was used with imputations made after every 500^th^ iteration following 1500 burn-in iterations to ensure that the imputations were independent. Ten completed datasets were imputed and the model of interest was fitted to each of these datasets. The results from the ten imputed datasets were combined using Rubin’s rule [29]. Analyses were conducted with {(males (n = 1018), females (n = 1163)} and without {(males (n = 768), females (n = 823), after listwise deletion of missing component scores and covariates} the cases with imputed results (Tables 3 &4). There were no meaningful differences in the results and therefore those for the entire sample are discussed in this paper.

**Table 3:** Regression coefficients (standard errors) of general practitioner and patient characteristics (number of general practitioners = 193) for physical components score

**Parameters (reference category)**	**Multilevel estimates for the main model**			
	**Male**		**Female**	
	**Complete cases**	**Imputed full model**	**Complete cases**	**Imputed full model**
	**(n = 768)**	**(n =1018)**	**(n =823)**	**(n =1163)**
*Fixed effects*				
Intercept	35.89	34.61	37.60	36.47
*Patient characteristics*				
Age, years				
40-59 (18–39)	−6.18 (2.44)*	−4.43 (2.39)	−2.87 (1.92)	−1.40 (1.60)
>59 (18–39)	−7.62 (2.47)^†^	−5.82 (2.41)*	−5.75 (1.96)^†^	−4.52 (1.62)^†^
Good or very good health (very bad, bad or fair health)	11.11 (0.69)^‡^	10.92 (0.75)^‡^	12.93 (0.70)^‡^	12.37 (0.67)^‡^
Own home & car (rented & don’t own a car)	2.14 (1.72)	2.79 (1.52)	4.07 (1.45)^†^	4.23 (1.38)^†^
Own home only (rented & don’t own car)	−0.83 (2.88)	−0.38 (2.98)	1.56 (1.69)	2.03 (1.65)
Own car only (rented & don’t own a car)	1.74 (1.83)	2.57 (1.72)	1.13 (1.63)	1.32 (1.57)
College / university (elementary / high school)	1.68 (0.73)*	1.65 (0.70)*	−0.19 (0.78)	0.05 (0.75)
Employed patients (unemployed^1^)	7.66 (1.15)^‡^	7.29 (1.04)^‡^	4.22 (0.99)^‡^	4.05 (1.08)^‡^
Retired patients (unemployed^1^)	3.90 (1.11)^‡^	3.86 (1.03)^‡^	0.09 (0.87)	0.08 (0.91)
Married/cohabiting (single/separated/divorced/widowed)	0.36 (0.88)	0.03 (0.87)	0.75 (0.75)	1.20 (0.72)
Two or more conditions (one condition)	−2.20 (0.68)^†^	−2.57 (0.69)^‡^	−1.79 (0.74)*	−1.56 (0.72)*
Smoker (non-smoker)	−0.54 (1.25)	−0.43 (1.23)	0.67 (1.14)	0.67 (1.13)
Overall satisfaction with care	0.07 (0.32)	0.13 (0.30)	−0.84 (0.33)*	−0.78 (0.38)*
*General Practitioner characteristics*				
Female (Male)	1.52 (0.98)	1.48 (1.04)	2.19 (0.80)^†^	1.59 (0.78)*
Overseas qualified (qualified in Australia)	1.39 (0.88)	1.37 (0.99)	0.47 (0.85)	−0.09 (0.83)
Years in the current practice 6–15 (< 6)	−0.27 (0.91)	−0.44 (0.93)	−0.80 (0.89)	−0.58 (0.85)
> 15 (< 6)	0.02 (0.90)	0.08 (0.92)	1.05 (0.95)	1.20 (1.05)
Years as a GP				
19–24 (< 19)	−0.80 (0.95)	−1.04 (0.96)	0.62 (0.89)	−0.43 (0.87)
> 24 (< 19)	0.61 (0.99)	−0.04 (1.0)	−0.57 (1.00)	−0.91 (1.01)
Size 1–3 GPs in the practice (4 or more GPs)	1.04 (0.72)	0.82 (0.81)	−1.54 (0.75)*	−1.10 (0.72)
Urban practice (Rural practice)	−0.18 (0.70)	−0.09 (0.69)	−0.27 (0.76)	−0.05 (0.74)
*Random effects*				
Variance between general practitioners (standard errors)	0.00 (0.00)	1.15 (1.60)	0.00 (0.00)	0.93 (1.56)
% variance explained	100	70.2	100	90.3
Variance between patients (standard errors)	81.84 (4.18)^‡^	84.66 (4.81)^‡^	91.49 (4.51)^‡^	93.29 (5.21)^‡^
% variance explained	38.6	36.3	37.4	36.3

Note:*P <0.05, ^†^ P <0.01, ^‡^ P<0.001.

^1^Includes patients looking for work, studying full-time, looking after family and unable to work due to sickness or disability.

Patient characteristics were collected independently using PACIC for the same respondents.

**Table 4:** Regression coefficients (standard errors) of general practitioner and patient characteristics (number of general practitioners = 193) for mental health components score

**Parameters (reference category)**	**Multilevel estimates for the main model**			
	**Male**		**Female**	
	**Complete cases**	**Imputed full model**	**Complete cases**	**Imputed full model**
	**(n = 768)**	**(n =1018)**	**(n =823)**	**(n =1163)**
*Fixed effects*				
Intercept	35.52	35.09	32.80	34.16
*Patient characteristics*				
Age, years				
40-59 (18–39)	0.48 (2.64)	0.31 (2.49)	2.83 (1.96)	2.42 (1.57)
>59 (18–39)	1.89 (2.68)	1.68 (2.51)	6.33 (1.99)^†^	5.62 (1.64)^‡^
Good or very good health (very bad, bad or fair health)	6.92 (0.75)^‡^	6.84 (0.79)^‡^	7.31 (0.71)^‡^	7.33 (0.62)^‡^
Own home & car (rented & don’t own car)	5.04 (1.87)^†^	3.42 (1.71)*	1.37 (1.48)	1.50 (1.29)
Own home only (rented & don’t own a car)	3.24 (3.12)	0.30 (2.77)	0.47 (1.72)	0.27 (1.61)
Own car only (rented & don’t own car)	3.04 (1.98)	1.55 (1.93)	0.67 (1.66)	0.67 (1.43)
College / university (elementary / high school)	0.78 (0.79)	0.65 (0.78)	0.07 (0.79)	0.00 (0.72)
Employed patients (unemployed^1^)	2.95 (1.24)*	3.40 (1.18)^†^	1.42 (1.01)	1.16 (1.03)
Retired patients (unemployed^1^)	4.95 (1.20)^‡^	5.43 (1.10)^‡^	2.62 (0.89)^†^	2.86 (0.87)^†^
Married/cohabiting (single/separated/divorced/widowed)	0.91(0.95)	0.96 (1.03)	2.29 (0.77)^†^	2.43 (0.73)^‡^
Two or more conditions (one condition)	0.11 (0.74)	−0.05 (0.77)	−0.96 (0.76)	−1.56 (0.73)*
Smoker (non-smoker)	1.62 (1.35)	1.16 (1.39)	−4.20 (1.17)^‡^	−3.99 (1.08)^‡^
Overall satisfaction with care	0.60 (0.35)	0.56 (0.36)	1.04 (0.34)^†^	0.84 (0.34)*
*General Practitioner characteristics*				
Female (Male)	0.55 (1.06)	0.41 (1.04)	−1.25 (0.81)	−0.74 (0.75)
Overseas qualified (qualified in Australia)	−1.87 (0.96)	−1.73 (0.98)	−0.28 (0.86)	0.02 (0.85)
Years in the current practice				
6–15 (< 6)	−1.32 (0.99)	−0.82 (0.97)	0.66 (0.91)	0.90 (0.88)
> 15 (< 6)	−0.04 (0.97)	0.02 (0.99)	0.18 (0.97)	0.36 (1.02)
Years as a GP				
19–24 (< 19)	0.35 (1.03)	0.51 (1.04)	0.88 (0.91)	0.49 (0.87)
> 24 (< 19)	0.58 (1.07)	0.44 (1.13)	0.23 (1.02)	−0.52 (1.04)
Size 1–3 GPs in the practice (4 or more GPs)	−0.84 (0.78)	−0.79 (0.80)	−0.63 (0.77)	−0.69 (0.69)
Urban practice (Rural practice)	0.87 (0.76)	0.93 (0.74)	−0.34 (0.78)	−0.32 (0.73)
*Random effects*				
Variance between general practitioners (standard errors)	0.00 (0.00)	0.92 (1.53)	0.00 (0.00)	0.30 (1.01)
% variance explained	100	84.3	100	88.1
Variance between patients (standard errors)	96.08 (4.90)^‡^	97.67 (5.35)^‡^	95.10 (4.69)^‡^	96.36 (4.43)^‡^
% variance explained	16.6	16.4	22.9	22.3

Note: *P <0.05, ^†^ P <0.01, ^‡^ P<0.001.

^1^Includes patients looking for work, studying full-time, looking after family and unable to work due to sickness or disability.

Patient characteristics were collected independently using PACIC for the same respondents.

## Results

The mean ages of male and female patients were 64 years (range 24–90) and 63 years (range 18–93) respectively.

The gender comparison for socio-demographic characteristics of patients and GPs is presented in Table 1. Female patients tended to have lower socio-economic status (13% more unemployed, 10% less owner-occupiers with cars, 6% more lower-educated) than male patients (Table 1; P < 0.001). Married patients were more likely to be males (78% vs 64%, P < 0.001). Male patients were more likely than females to have more than one chronic disease (P < 0.001). Female GPs were more likely to have female than male patients (33% vs. 15%, P < 0.001) and the proportions were 37% vs. 18% (P < 0.001) without solo practices. Female patients tended to be under the care of younger (practicing for < 19 years) GPs than male patients (37% vs 29%, P < 0.001).

The overall means of PCS-12 scores for male and female patients were 43.0 (SD = 11.7) and 42.3 (SD = 12.4) respectively (Table 2). Similarly, overall MCS-12 scores were 50.0 (SD = 10.9) for men and 48.3 (SD = 11.2) for women. Table 2 shows the differences between unadjusted PCS-12 or MCS-12 scores of male and female patients for the subcategories of patient and GP characteristics (Table 2). Women reported poorer physical health than men among those who had one chronic disease (P = 0.035) and were under the care of a male GP (P = 0.014, Table 2). Unemployed females reported better physical health than unemployed males (P = 0.020). Similarly, women reported poorer mental health than men across the following patient characteristics: middle age (P = 0.005), home and car owners (P = 0.007), employed (P = 0.003), two or more chronic diseases (P = 0.01), smokers (P = 0.001), high or low PACIC scores (P = 0.03), under the care of a male GP (P = 0.017), a GP who was Australian trained (P = 0.001), < 6 (P = 0.028) or > 15 years (P = 0.003) as a GP in the current practice, > 24 years as a GP (P = 0.013), who worked in a larger practice (P = 0.006) or in an urban area (P < 0.001) (Table 2). However, the above effects were not adjusted for confounding effects.

Gender differences in independent variables after adjustment for confounding effects with the multilevel regression analyses for each of the response variables are presented in Tables 3 and 4.

PCS-12 scores declined with age for both male (P < 0.05) and female patients (P < 0.01), but in contrast MCS-12 scores increased with age for female patients (P < 0.001).

Both men and women with good general health were likely to have clinically significant better physical health with large effect sizes (> 1.3, B (regression coefficient) > 10.9, P < 0.001) and better mental health with a moderate effect size (> 0.7, B > 6.8, P < 0.001) compared to those with poor general health (Tables 3 &4). Home and car ownership was positively associated with PCS-12 scores of female patients (effect size = 0.79, B = 4.2, P < 0.01) and MCS-12 scores of male patients (effect size = 0.82, B = 3.4, P < 0.05). Patients who were employed (effect size = 1.23, B = 7.3, P < 0.001) or retired (effect size = 0.51, B = 3.9, P < 0.001) were likely to have higher PCS-12 scores and MCS-12 scores (effect size > 0.55, B > 3.4, P < 0.01) than unemployed male patients. Employment was likely to have a positive effect on PCS-12 scores (effect size = 0.78, B = 4.1, P < 0.001) and retirement was likely to have a positive effect on MCS-12 scores (effect size = 0.52, B = 2.9, P < 0.01) for women. An effect size of more than 0.5 is considered to be clinically significant [3]. The number of chronic medical conditions was negatively associated with both MCS-12 (P < 0.05) and PCS-12 scores (P < 0.05) for women and with PCS-12 (P < 0.001) for men. Female patients who were married or cohabiting tended to have higher MCS-12 scores than those who were not (P < 0.001). Female smokers were likely to have a lower mental health score than non-smokers (effect size = 0.66, B = −4.0, P < 0.001). Neither smoking status nor marital status was associated with PCS-12 scores for any gender or MCS-12 scores for men. Results also showed a positive association between patient assessed quality of care and MCS-12 scores (P < 0.05) and a negative association between quality of care and PCS-12 scores (P < 0.05) for females only. Effect sizes for some of the significant effects (for example, number of conditions, satisfaction with care, married status) were small (< 0.5) and clinically not significant. Being under the care of a female GP was likely to have a positive effect on PCS-12 scores (P < 0.05) of female patients. Other GP characteristics were not associated with either PCS-12 or MCS-12 scores for either gender.

### Variance explained

At the GP level (level 2), 70% and 90% of the variances in PCS-12 scores between GPs for male and female patients were explained respectively by the variables used in the analysis (Table 3). At the patient level (level 1) the variance in PCS-12 scores explained was similar (36%) for male and female patients (Table 3). Similarly, for MCS-12 scores, GP level variances explained were 84% for males and 88% for females and patient level variances explained were 16% for males and 22% for female patients (Table 4).

## Discussion

This study is one of very few to explore gender differences in HRQoL among patients with type 2 diabetes and hypertension/ischaemic heart disease. The SF-12 is a subjective measure of health that can be influenced by a respondent’s perceptions, expectations and interpretations about health [6]. Nonetheless, the scale has become one of the most widely used HRQoL measures. This study provides comprehensive data on how patient and GP characteristics predict self-rated physical and mental health of chronically-ill patients in Australia.

The results show that chronically-ill women smokers reported clinically significantly (effect size = 0.66) poorer mental health than non-smokers. This supports findings from previous studies showing that the association between smoking and HRQoL is different between women and men [13,30,31]. Lasser et al. [10] suggested that people with poor mental health are more likely to smoke than those who have good mental health. A higher physical and psychological dependence among women is a possible explanation for the increased mental distress observed among women who unsuccessfully attempted to quit [32]. Strine et al. [8] found a significant association between smoking and impaired mental health. The provision of psychological care in conjunction with smoking-cessation programs, and vice versa, is indicated [33]. Smoking is one of the strongest modifiable risk factors for a host of health outcomes that contribute to morbidity and mortality in Australia and worldwide in chronically-ill patients. All this suggests that chronically-ill women may need more psychological support in their attempts to quit smoking. Smoking is one of the most significant risk factors for the development of cardiovascular disease in diabetes patients [34]. Smoking is a risk factor for mortality and coronary heart disease in hypertension and in diabetes [35]. Chronically ill, particularly females with mental illness are motivated to attend smoking reduction and cessation programmes. When supported programmes providing nicotine replacement and counseling are offered to people with mental illness, it has been found that they are able to quit smoking at equivalent rates to the general population [36]. It is important to ensure that the person with severe mental illness is aware of the risks of smoking because basic medical education is frequently missing in this patient group [37]. Integrated care is desirable, as psychiatric symptoms may be exacerbated and severe withdrawal symptoms experienced by smokers with a mental illness undertaking smoking cessation treatment. When smoking cessation and psychiatric care is integrated these adverse effects appear to be reduced [38]. The ability to detect hypothesized relationship in previous studies between smoking and mental health of females suggests construct validity of smoking status in this study.

The results show that women under the care of female GPs reported better physical health (effect size = 0.30) than those under the care of male GPs. Physician’s gender is one of many factors that impact on the doctor patient interaction [16]. As noted in previous studies, female GPs tend to have longer consultations [15,39], especially with their female patients. Female patients often report that they prefer female doctors for female-specific health problems [40,41], intimate problems [42,43], behavioral problems [41], and endocrinologic/metabolic problems [14]. Female physicians were found to be more likely to perform female-specific prevention procedures, check patients’ blood pressure and make some follow-up arrangements and referrals [44]. Also, compared to their male colleagues, female physicians were shown to use a more participatory decision-making process [45] that encourages self-management, which is a fundamental part of diabetes care and may result in improved diabetes control [46].

Socio-demographic differences between male and female patients in the current study (e.g., the women were younger, less likely to be married, and had lower socio-economic status than the men) are consistent with those found in other similar studies [47,48] and provide an understanding of gender differences in HRQoL.

Our study showed that there are gender differences in how patient characteristics impact on self-assessed physical and mental health. For example, previous research found that lower socio-economic groups reported lower PCS-12 and MCS-12 [4,5,49]. Our results showed that home and car ownership tended to have a positive effect on the self-assessed physical health of women but not men and on the mental health of men, but not women. Some studies have shown a significant interaction effect between gender and employment, indicating that employed men enjoyed higher levels of general well-being [2,7,50]. In this study the negative impact of unemployment was likely to be greater in male than female patients. Unemployment was likely to have a larger negative effect on HRQoL of men than that of women. This may be because the significance of work and its impact on household income may be greater in chronically-ill older men than in women [51]. Also the observed age difference in HRQoL differed among men and women. The younger men (< 39 years) were likely to report better physical health than the older men (> 59 years), whereas the older women reported better mental health than the younger women.This result is consistent with previous studies. For example, Hanmer et al. [52] reported similar mental health (MCS-12) mean scores for younger men (< 39 years, 52.1) and older men (> 59 years, 52.3) whereas older women reported better mental health mean scores (51.3) than younger women (49.6).

Two hundred and eleven patients suffered from only type 2 diabetes and 793 from type 2 diabetes and hypertension and/or ischemic heart disease. Further 1129 suffered from hypertension and/or ischemic heart disease. The overall mean PCS-12 of male diabetes only patients (46.7, SD = 12.4) and male hypertension/ischaemic heart disease only patients (44.8, SD = 11.5) in the study were less than male U.S. general population (mean = 50.6, SD = 10.2) [19]. The difference for PCS-12 was clinically not significant for diabetes (effect size =0.38 < 0.5), whereas it was clinically significant (effect size = 0.56) for hypertension/ischaemic heart disease. Similarly, the overall mean PCS-12 of female diabetes only patients (44.7, SD = 11.6) and female hypertension/ischaemic heart disease patients (43.4, SD = 12.6) were less than female U.S. general population (mean = 48.7, SD = 9.6) [19]. The difference was clinically not significant for diabetes (effect size =0.41), whereas for hypertension/ischaemic heart disease patients, the difference was clinically significant (effect size = 0.53). However, mean MCS-12 of male (49.3, SD = 10.6) and female (47.7, SD = 12.3) diabetes only patients and male (50.5, SD = 10.5) and female (49.2, SD = 10.8) hypertension/ischaemic heart disease patients was not clinically significant compared to male (50.4, SD = 9.9) and female (48.4, SD = 9.6) MCS-12 scores of U.S. general population.

Although the GP level variance explained was very high (from 70% to 90%) for both summary scores, the patient level variance explained for mental health was half that of physical health (36%). There may have been other lifestyle and clinical risk factors important to mental health assessment which were not specifically evaluated in this study and warrant further exploration in the Australian context.

There are a number of limitations to this study. Patients that the practice identified as being unable to read English were excluded from the study. Although the response rate of 70% was comparable with other studies [53], it is possible that non-responders might have assessed their physical and mental health differently from those who responded. We were unable to analyse differences between respondents and non-respondents in the study as information about non-respondents could not be captured as recruitment was at arm’s length through practices. In a similar HRQoL study in Australia with 7606 chronically-ill patients from 96 general practices, the response rate was 61% [2]. In that study we had gender for non-respondents. We conducted analyses comparing proportions of respondents with non-respondents for gender. The gender of respondents (53.3% were females) and non-respondents (53.6% were females) were similar (P = 0.76). As females attend a GP more often than males, they have a greater chance of being selected in the sample [54]. Male (46.7%) and female (53.3%) patients responded to mental health questions in the study were similar to other studies [2]. Further, the socio-economic status of male patients were similar in both studies (home owners: 82.3% vs. 81.4%). We compared patient characteristics of this study with a similar Australian general practice study with type 2 diabetes, ischaemic heart disease/hypertension and asthma patients [55]. The proportions of gender (P = 0.712), home ownership (P = 0.690), and marital status (P = 0.903) were similar between the two studies (data not shown). However, the patients in this study were older (69% vs. 55% for > 59 years, P < 0.01) and marginally less employed (32% vs. 34%, P = 0.024). The reason being that, in the other study asthma patients (n = 724) were much younger (mean = 50 years) and our study does not have any asthma patients. The mean age in our study was 64 years. Hence our study had more older and retired (47% vs. 40%), and therefore less employed patients.

In Australia, the proportion of males and females who smoked declined 1.4% (22.5% in 2004 and 21.1% in 2007) and 1.1% (18.8% in 2004 and 17.7% in 2007) respectively between 2004 and 2007 [56]. In Australia, the proportion of males and females type 2 diabetes and IHD patients who smoked in 2007 were 13.9% and 15.3% respectively [57]. Those figures were slightly higher than the proportions for type 2 diabetes and/or IHD patients for males (9.2%) and females (11.1%) of our study.

There were no significant differences in GP characteristics between our sample and all GPs in Australia in terms of female gender (37.3% vs. 35.9%). This proportion was similar in Beech study (36.8%) [54]. However, GPs who graduated in a country other than Australia were slightly under-represented when compared with Australian total sample (25.5% vs. 30.2%) but not with those participated in Beech study (26.5%) [54]. In Australia, patients can choose what doctor they see, at whichever practice they choose. There are no patient boundaries and patients do not have to ‘sign up’ with a particular practice.

The actual ICC computed for MCS-12 from the final multilevel model is 0.003 for females and 0.009 for males which is lower than that used in the power calculations. With actual ICC, 960 patients from each gender were adequate to detect an effect size of 0.13 between smokers and non-smokers.

Strengths of the study include the large number of patients and GPs participating, the adjustment for confounding patient and GP factors, the correction for GP-level clustering with multilevel modeling, and addressing the potential bias and loss of precision arising from missing values using multiple imputations.

## Conclusions

The increased prevalence of poorer self-assessed mental health status among chronically ill women who smoke is deserving of further investigation. Better understanding of the associations between HRQoL and smoking may help to reduce barriers to smoking cessation and help to better tailor smoking cessation support to patients’ needs, ultimately improving their overall well-being. This is especially important in the context of chronic disease, where improvement of lifestyle behavior is central to management. Clinicians are advised to pay more attention to the mental health status of chronically-ill smokers, specially women.

More quantitative studies are needed to explore the interaction between GP gender and patient gender in relation to HRQoL.

## Abbreviations

B: Regression coefficient; CCI: Clinical care interview; BP: Bodily pain; GH: General health; GP: General practitioner; HRQOL: Health-related quality of life; ICC: Intra-cluster correlation; MCS: Mental Health Component Summary; MCS-12: Mental Component Score derived from the SF-12; MH: Mental health; PACIC: Patient Assessment of Chronic Illness Care; PCS: Physical Component Summary; PCS-12: Physical Component Score derived from the SF-12; PF: Physical functioning; RE: Role emotional; RP: Role physical; SD: Standard deviation; SF: Social Functioning; SF-12: Short Form 12-item Health Survey; SF-36: Short Form 36-item Health Survey; VT: Vitality.

## Competing interests

The authors declare that they have no competing interests.

## Authors’ contributions

UJ contributed to data analysis, interpreting the data and drafting the manuscript. UJ and MH made substantial contributions to the conception and design of the study. JT and BC were involved in the data collection. All authors were involved in drafting the manuscript or revising it critically for important intellectual content. All authors have read and approved the final version of the manuscript.
